# Environmental and Household-Based Spatial Risks for Tungiasis in an Endemic Area of Coastal Kenya

**DOI:** 10.3390/tropicalmed7010002

**Published:** 2021-12-23

**Authors:** Ayako Hyuga, Peter S. Larson, Morris Ndemwa, Sheru W. Muuo, Mwatasa Changoma, Mohamed Karama, Kensuke Goto, Satoshi Kaneko

**Affiliations:** 1 Graduate School of Biomedical Sciences, Nagasaki University, 1-12-4 Sakamoto, Nagasaki-shi 852-8523, Nagasaki, Japan; ayako.hyuga@gmail.com; 2 Department of Eco-Epidemiology, Institute of Tropical Medicine, Nagasaki University, 1-12-4 Sakamoto, Nagasaki-shi 852-8523, Nagasaki, Japan; morrisndmw@gmail.com; 3 Nagasaki University Institute of Tropical Medicine-Kenya Medical Research Institute (NUITM-KEMRI) Project, C/O Centre for Microbiology Research, KEMRI, Nairobi P.O. Box 19993-00202, Kenya; anfangen@umich.edu (P.S.L.); sheruwanyua@yahoo.com (S.W.M.); jtasa06@gmail.com (M.C.); 4 Social Environment and Health, Survey Research Center, Institute for Social Research, University of Michigan, Ann Arbor, MI 48109, USA; 5 Department of Epidemiology, School of Public Health, University of Michigan, Ann Arbor, MI 48109, USA; 6 Centre of Public Health Research, Kenya Medical Research Institute (KEMRI), Off Mbagathi Road, Nairobi P.O. Box 54840-00200, Kenya; mkarama@umma.ac.ke; 7 Division of Health and Safety Sciences Education, Department of Educational Collaboration, Osaka Kyoiku University, 4-698-1 Asahigaoka, Kashiwara-shi 582-8582, Osaka, Japan; goto@cc.osaka-kyoiku.ac.jp

**Keywords:** tungiasis, spatial epidemiology, Kenya, Health and Demographic Surveillance System, generalized additive models, zoonosis, parasitosis, global health, diseases of poverty, GIS

## Abstract

Tungiasis is a cutaneous parasitosis caused by an embedded female sand flea. The distribution of cases can be spatially heterogeneous even in areas with similar risk profiles. This study assesses household and remotely sensed environmental factors that contribute to the geographic distribution of tungiasis cases in a rural area along the Southern Kenyan Coast. Data on household tungiasis case status, demographic and socioeconomic information, and geographic locations were recorded during regular survey activities of the Health and Demographic Surveillance System, mainly during 2011. Data were joined with other spatial data sources using latitude/longitude coordinates. Generalized additive models were used to predict and visualize spatial risks for tungiasis. The household-level prevalence of tungiasis was 3.4% (272/7925). There was a 1.1% (461/41,135) prevalence of infection among all participants. A significant spatial variability was observed in the unadjusted model (*p*-value < 0.001). The number of children per household, earthen floor, organic roof, elevation, aluminum content in the soil, and distance to the nearest animal reserve attenuated the odds ratios and partially explained the spatial variation of tungiasis. Spatial heterogeneity in tungiasis risk remained even after a factor adjustment. This suggests that there are possible unmeasured factors associated with the complex ecology of sand fleas that may contribute to the disease’s uneven distribution.

## 1. Introduction

Tungiasis is a zoonotic, cutaneous parasitosis caused by the embedding of female sand fleas (*Tunga* spp.) into the upper strata of the skin [1]. Tungiasis is common to poor communities in sub-Saharan Africa, South America, and the Caribbean [2]. The female flea burrows headfirst into the upper strata of the skin. After copulation, the female produces eggs, which cause the flea to expand to several times its original size, compressing and causing stress to the surrounding skin tissues. Eggs are expelled through the exposed posterior, fall to the ground, and mature in sands and soils [3,4].

Tungiasis represents the classic neglected tropical disease: it afflicts the poorest people mainly in very rural communities in tropical countries, and there is little research and almost no investment in efforts to prevent, control, and/or treat the disease [1]. Few studies have reported on affected populations globally, though over 20 million in the Americas and 668 million people in sub-Saharan Africa are estimated to be at risk of tungiasis [5,6].

To prevent and control tungiasis, it is essential to elucidate specific risk factors that raise or mitigate infection. Several environmental, demographic, and behavioral factors have been associated with disease incidence. These include age groups, specifically children and the elderly [7,8,9,10,11]; low socioeconomic status associated with inadequate housing [7,9,11,12,13,14,15,16]; low affordability, access to, and knowledge of hygiene and health behaviors [11,12,14,15]; and the lack of clean water and sanitation at the household level [7,16]. Relevant to the zoonotic aspects of tungiasis, the presence of animals of various species in and around household compounds was also reported to raise the risk of human tungiasis [9,11,12,13,14,17]. These risk factors are also common to poor communities in tropical developing countries. More detailed information on how risk for tungiasis is distributed within communities is needed for the efficient development and implementation of preventive measures.

The geographical distribution of tungiasis can vary widely, even between neighboring areas that share similar risk profiles [1,7,16]. The environmental suitability of tungiasis varies across the sub-Saharan African continent, and comprises a complex range of topographic, environmental, and human-influenced factors (e.g., livestock density) [6]. In particular, soil suitability for sand fleas may influence their distribution, as the soil is the site of their off-host development [3,18]. Therefore, we should simultaneously consider environmental and human risk factors in the analysis for tungiasis infection and the recorded geographic distribution of tungiasis infection when assessing areas of risk. To date, however, there have been few epidemiological studies of tungiasis that consider how potential environmental factors and risk factors related to individuals and households determine the spatial distributions of the disease using on the ground, house to house prevalence surveys in sub-Saharan Africa.

This research tested three main hypotheses. First, we suspect that tungiasis risk will be heterogeneously distributed, i.e., households at a high risk for tungiasis will be proximal to other households at risk. Second, we suspect that environmental factors, such as soil type, wetness, and proximity to a wildlife reserve, will explain the spatial variation of tungiasis cases in this area. Third, we speculate that a combination of environmental and household factors will work in concert to raise or lower risk.

This study aims to address spatial risks that cause a spatially heterogeneous distribution of tungiasis by considering risk factors related to demographics and socio-economy, as well as spatial ecological factors, in a tungiasis endemic area using a comprehensive, household-level tungiasis survey in a resource-poor, rural area of East Africa.

## 2. Materials and Methods

### 2.1. Study Population and Area

**Figure 1:**
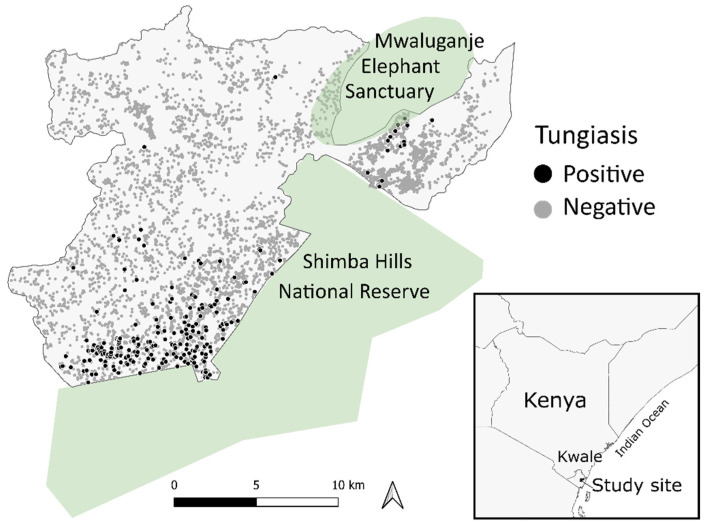
Distribution of the households with or without tungiasis in the Kwale-HDSS area.

Tungiasis infection data and household information were collected by the Health and Demographic Surveillance System (HDSS) managed by the Nagasaki University Institute of Tropical Medicine and Kenya Medical Research Institute (NUITM-KEMRI) [19]. The HDSS conducts survey activities in Kwale County, along the southern coastal region of Kenya on the Indian Ocean. Kwale is an arid area that belongs to the savanna climatic zone. The HDSS in the study area was set in 2010 to monitor the population dynamics of around 40,000 residents (10,000 households), such as births, deaths, and migrations, in the coverage area of approximately 385 km^2^, where the geographical location ranges between 5°42′52″ S, 39°14′35″ E and 5°55′31″ S, 39°31′6″ E along the northern side of the Shimba Hills National Reserve and surrounding the Mwaluganje Elephant Sanctuary (Figure 1) [19]. All residents enrolled in the HDSS who were present at home at the time of data collection and who consented to participate were included in the analysis.

- Inclusion and exclusion criteria

All permanent residents of the HDSS area over the age of nine months were considered eligible for inclusion in the study. Non-permanent residents, defined as people not intending to stay in the HDSS area for more than three months, are not normally included in routine data collections and thus were not included in the study.

### 2.2. Tungiasis Infection and Household Information

We used survey information on tungiasis infection collected between March and December 2011 during the HDSS follow-up survey. Survey teams were trained by clinical officers to identify signs and symptoms of tungiasis. At the time of data collection, cases were identified by first asking individuals if they were experiencing a tungiasis infection. If the individual reported that he or she was infected, either through self-report or through a household representative, the case status was confirmed by visual inspection. Household members who were not reported as being infected were considered negative and recorded as such. No attempt was made to grade lesions using an assessment tool, such as the Fortaleza classification [4], and to identify the species of *Tunga* present in participants’ lesions. Our ethical approval did not allow for the invasive extraction of gravid female sand fleas from the skin, which is necessary for species identification [20].

In the case of data duplication as a result of visiting the household more than once, information from the first time the individual was surveyed was retained for analysis. This study considered household-level infection, rather than individual infection, when assessing spatial risks. We recorded households as “tungiasis present” when at least one individual in the home was positive for tungiasis. This binary measure was used as an outcome in all analyses for this research.

Household-level explanatory variables included latitude/longitude coordinates, wall, floor, and roof materials, and toilets. These were available from the HDSS baseline survey conducted in July 2010 and from follow-up surveys through 2012. Households with missing information were excluded from the analysis.

Household demographic information composition was aggregated to the household level. We calculated the numbers of children under 15 years of age and elderly over 60 years of age per household to assign the age structure of household members. For those who belonged to more than one household (e.g., polygamy or second house owner), we assigned a value divided by the number of households they belonged to (e.g., 0.5 people if a person belongs to two households) as the number of residents per household (number of people per household). After conversion, households with more than 19 people (upper quartile plus three quartile ranges) were excluded as outliers.

### 2.3. Ecological Factors

We included several variables related to the ecology of *Tunga* spp.; specifically, we chose variables relevant to off-host stage development based on a review of relevant literature [3,6,12,21]. The data comprised remote sensing and freely available data sets. See Table S1 for details. Pointwise values were extracted using latitude/longitude coordinates of each household. All raster to point data extraction was conducted on QGIS 3.14 [22]. The following is a brief description of the environmental data used in this study.

#### 2.3.1. Normalized Difference Vegetation Index (NDVI) and Yearly Land Cover in 2011

NDVI data were obtained from the United States National Aeronautics and Space Administration Land Processes Distributed Active Archive Center (NASA LP DAAC) through the Earth Engine Data Catalog [23]. The median NDVI of each pixel was calculated from merged layers of 16-day NDVI with masking low-quality pixels. Then, NDVI was integerized using the following equation: NDVI (8 bit)=(NDVI+1)×100. The generated value ranges from 0–200 instead of the original range, which is from −1 to 1.

#### 2.3.2. Topographic Wetness Index (TWI)

TWI is a proxy for soil wetness index, representing where water will accumulate, taking elevation differences into account [24,25]. Source data to compute TWI, the Advanced Spaceborne Thermal Emission and Reflection Radiometer (ASTER) Global Digital Elevation Model (GDEM), was obtained from the Ministry of Economy, Trade, and Industry (METI) of Japan and NASA through Earthdata Search [26]. Required components (catchment area, flow width, and slope) calculating the TWI were computed using recommended algorithms [25]. Initially, terrain sinks were removed from the Digital Elevation Model data using Sink Removal with Fill Sinks in SAGA GIS 2.3.2. Then, the total catchment area was calculated by the Multiple Flow Direction algorithm [27] with the convergence at 1.0. Secondly, the slope was calculated in the algorithm of 10 parameters and third-order polynomial [28]. TWI was lastly computed using the pseudo-specific catchment area.

#### 2.3.3. Elevation Data

Elevation data were obtained from Advanced Land Observing Satellite (ALOS) World 3D–30 m provided by the Japan Aerospace Exploration Agency (JAXA) [29].

#### 2.3.4. Soil Properties

Data on soil properties were obtained from the International Soil Reference and Information Centre (ISRIC) [30]: Soil pH in H_2_O at a depth of 0 cm [31], soil texture at a depth of 0 cm [31], soil organic carbon content [31], the extractable aluminum content of the soil at a depth of 0–30 cm [32], and the extractable iron content of the soil at a depth of 0–30 cm [32]. Layer data closest to a depth of 5 cm, where sand flea larvae exist, were chosen [3]. *Tunga* spp. are found in sandy, clay, or laterite soils [18,33]. Many descriptions can be found for soil texture, such as sandy and clay soils, but there is little for laterite [2,9,11,12,34,35]. Therefore, the latter two mineral contents characterizing laterite were included in the exploratory analysis.

#### 2.3.5. Distance from Households to the National Reserve or the Elephant Sanctuary

As wild animals in the Shimba Hills National Reserve and the Mwaluganje Elephant Sanctuary might have played a potential role in transmitting tungiasis around the buffer zone, Euclidean distance from households to the nearest reserve was calculated in QGIS 3.14.

### 2.4. Statistical Analysis

Generalized additive models (GAMs) [36] were used to create a predictive map of odds ratios (ORs) of household tungiasis infection over the study area. They were also used to estimate ORs for other risk factors. We used the MapGAM [37] and the gam [38] packages in R version 4.0.3 [39]. GAMs are extensions of typical linear regressions models (e.g., logistic regression model for binary data) that include smoothed terms that allow for the relaxing of typical assumptions of linearity between predictors and outcomes [40]. In the field of spatial epidemiology, mixed-mode GAMs, in which only location coordinates are non-parametrically smoothed, whereas other covariates keep a form of linear parametric prediction [36], have been used to investigate the geographic variation of diseases [36,41,42,43,44,45,46,47]. The below model was used to calculate ORs in this study:

$$\log\left(\frac{p_{i}}{1-p_{i}}\right)=S\left(x_{i},\;y_{i}\right)+Z_{i}\beta +\alpha$$

where *p_i_* denotes the probability that the *i*th household has one or more tungiasis cases; *x_i_* and *y_i_* are projected coordinates for the *i*th household; *S*(*x_i_*, *y_i_*) is a 2-dimensional smoothing of location; *Z_i_* is a vector of covariates for the *i*th household (household-level variables); *β* is a vector of regression parameters; *α* is an intercept [36].

A locally weighted scatterplot smoother (loess) was used for two-dimensional smoothing. A span size, specifying a percentage of neighborhood data points used for smoothing, was determined by minimizing the Akaike information criterion [36]. Regular grid points used for predictions were created using a shapefile of the HDSS area in the same projection as the household’s coordinates. The numbers of rows and columns of the grid were adjusted to make square grids rather than rectangle ones by reflecting the east-west and north-south distances. A function, modgam in the MapGAM package, fit a GAM model and estimated the log-odds at each of the grid points. The OR was calculated by the odds at each grid point and the median log odds over the study area [48]. To test the global null hypothesis that geolocation of household is unassociated with tungiasis occurrence, we conducted a permutation test with 1000 conditional permutations for a global *p*-value. As the conditional permutation test with a fixed span size induces an inflated type I error rate, the alpha risk was set to be 0.025 so that the type I error rate was within 0.05 [49]. At the same time, a pointwise permutation test was conducted to determine areas with significantly increased or decreased odds (hot spots and cold spots) [36]. The permutation distribution was calculated at each grid point using the same permutation data set for the global test (*p* = 0.05). Significantly increased or decreased OR area was defined as an area with grid points that rank in the higher 97.5% or the lower 2.5% of the pointwise permutation distribution, and contour lines of 0.975 and 0.025 indicate increased or decreased OR area, respectively [36]. The distribution pattern of the disease was recognized by significant hot and cold spots.

Spatial risk factors were examined by comparing a risk surface of a map between the unadjusted and an adjusted model. First, each variable was added to the unadjusted model to determine whether it was a spatial risk or not, as previously mentioned [42,46]. Then, variables that changed the surfaces by 10% were added to the unadjusted model together [42,46]. If the odds ratio increased or decreased by 10% from the unadjusted model at any grid point in the adjusted model, the adjusted variable was considered a spatial risk. As a result, all variables showed a risk surface change of more than 10%, and they were included in models as follows. In an adjusted model (model 1), we included the following: the number of children per household, the number of elderly per household, the number of males per household, type of floor (earthen/non-earthen), type of wall (earthen/non-earthen), type of roof (organic/non-organic), type of toilet (flush or pit latrine/none), NDVI, land cover (grasslands/croplands/savannas), TWI, elevation, soil pH, soil texture (sandy clay loam/sandy loam), soil organic carbon content, aluminum content in the soil, and iron content in the soil. In another adjusted model (model 2), we added the distance to the nearest animal reserve area to explore the effect of the distance on tungiasis risk. Models were assessed for multicollinearity by the variance inflation factor using a reduced model (i.e., without smoothing of location).

## 3. Results

### 3.1. Demographic Characteristics

Among 41,618 participants where tungiasis status was known, 483 individuals were excluded due to missing information, and 41,135 individuals comprising 7925 households were retained and used for analysis. A total of 461 people (1.1%) from 272 households (3.4%) had tungiasis at the time of the data collection. See Figure 1 for the scatter of the tungiasis positive and negative households. The age of participants ranged from one to 113 years (mean = 22 years), and the gender was almost equal: 50.7% female and 49.3% male. The data collection extended over both the dry and rainy seasons: 12.0% in March, 35.2% in April, 27.4% in May, 17.2% in June, 2.4% in July, 2.7% in August, 2.8% in September, 0.2% in November, and 0.1% in December. More than half of the data were collected in the rainy season in April, May, November, and December.

### 3.2. Spatial Analysis

**Figure 2:**
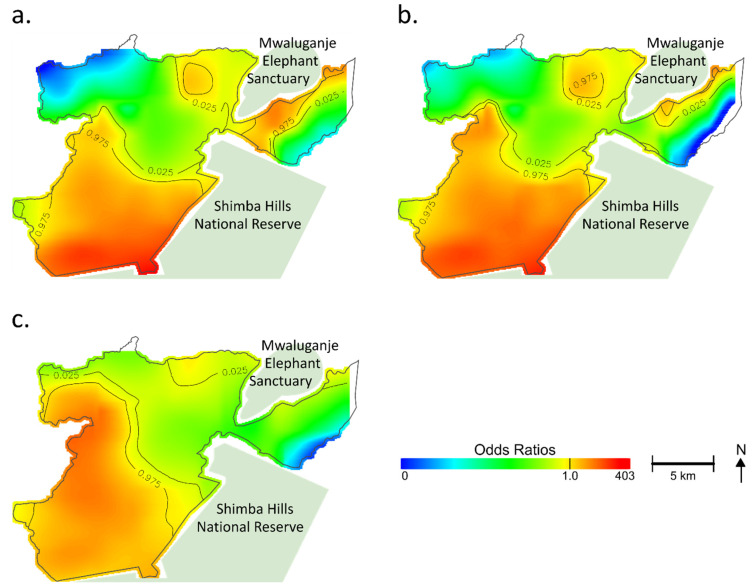
Geographical distribution of estimated odds ratios of tungiasis in the household. For easy comparison, a color band is fixed in an arbitrary range. Regions at the edge of the study area and no data or regions with odds ratios (ORs) outside the fixed range are shown in white. Based on the permutation test, contour lines indicate areas with significantly increased or decreased ORs. (**a**) Unadjusted; (**b**) Model 1 adjusted for the number of children per household, the number of elderly per household, the number of males per household, type of floor, type of wall, type of roof, type of toilet, Normalized Difference Vegetation Index (NDVI), land cover, Topographic Wetness Index (TWI), elevation, soil pH, soil texture, soil organic carbon content, aluminum content in the soil, and iron content in the soil; (**c**) Model 2 adjusted for the number of children per household, the number of elderly per household, the number of males per household, type of floor, type of wall, type of roof, type of toilet, NDVI, land cover, TWI, elevation, soil pH, soil texture, soil organic carbon content, aluminum content in the soil, iron content in the soil, and distance to the nearest animal reserve area.

In the unadjusted analysis, the variation of ORs displayed a pattern that corresponded with the geographical distribution of households with positive cases (Figure 2a). The optimal span size that minimized the AIC was 0.1, meaning that 10% of neighboring data were used for smoothing. Most areas had significantly increased or decreased ORs. A *p*-value for the global permutation test was less than 0.001 (<0.025: *α*), indicating that the occurrence of tungiasis was significantly associated with the geolocation of the household. Significantly increased ORs were observed among households in the east and west sides of the Mwaluganje Elephant Sanctuary and the southern part centering on the region convex southeastward (Figure 2a). In contrast, households in the northwest and northeast showed significantly decreased ORs.

Figure 2b shows the adjusted map (model 1) for the spatial risks. While the southern part still showed significantly increased ORs, there was a shrinking of areas at increased ORs on the east side of the Mwaluganje Elephant Sanctuary (See Figure 2b). The heterogeneous distribution of the selected variables (e.g., spatial aggregation of risks) partially explained the geographic distribution of hot spots of tungiasis. However, even after adjustment, a significant association between location and tungiasis remained (global *p*-value < 0.001), and the adjusted map did not become fully flat (Figure 2b). The optimal span size was 0.1 in model 1.

Figure 2c shows model 2, which was additionally adjusted for the distance to the nearest animal reserve area to see the additional impact. The optimal span size was 0.15 in model 2. The centering of the hot spot moved from the convex (Figure 2b) to the west nook (Figure 2c), reflecting the adjustment for the distance to the nearest animal reserve area. However, the southern region still had increased ORs, and the unexplained geographical risk variance remained (global *p*-value < 0.001). The variance inflation factors of the models were less than ten, indicating that multicollinearity might not have existed. No variables were excluded from the analysis.

Table 1 shows the used factors in the multivariate GAMs. In model 1, the number of children per household was associated with tungiasis (OR: 1.4, 95%CI: 1.3–1.5) at a host-related level. Earthen floors (OR: 3.2, 95%CI: 1.4–7.7) and organic roofs (OR: 1.7, 95%CI: 1.1–2.8) significantly affected the tungiasis status in socioeconomic factors. A positive association was observed among ecological factors at the household point in elevation (OR: 1.2, 95%CI: 1.1–1.3), aluminum content (OR: 1.1, 95%CI: 1.03–1.1), and iron content (OR: 1.2, 95%CI: 1.01–1.5). Although insignificant, indexes related to greenness and wetness, NDVI, and TWI showed positive associations with tungiasis. In model 2, the distance to the nearest animal reserve area was also significantly associated with tungiasis (OR: 0.6, 95%CI: 0.5–0.7). One kilometer away from the nearest animal reserve area, the OR was reduced to almost half. On the other hand, the iron content was not a significant spatial risk through an additional adjustment for the distance to the nearest animal reserve.

## 4. Discussion

This study revealed both the variability in the spatial distribution of tungiasis cases in a tungiasis-endemic area and the risk factors for tungiasis by adjusting for the spatial distribution of the potential risk factors. The pointwise permutation test found that the distribution of the disease was heterogeneous, with some areas having significantly increased or decreased ORs. The significance remained even after adjusting for other covariates: the number of children per household, the number of elderly per household, the number of males per household, type of floor, type of wall, type of roof, type of toilet, NDVI, land cover, TWI, elevation, soil pH, soil texture, soil organic carbon content, aluminum content in the soil, iron content in the soil, and distance to the nearest animal reserve area. Risk factors identified in this study were the number of children in the home, earthen floor, organic roof, elevation, aluminum content in the soil, and distance to the nearest animal reserve.

Our results support other research and have many implications for the complex network of factors that determine the household risk for tungiasis. First, an increased number of children in the home, earthen floor, and organic roof were associated with an increased spatial risk for household tungiasis risk, which agrees with the results of numerous studies on tungiasis [7,8,9,10,11,12,13,14,15,16]. Children spend much of their time on bare feet, especially in poor rural areas, which increases the chance of infection and transmission of tungiasis. Thus, it is more likely for tungiasis to be maintained in a household as a result of having more susceptible hosts. Organic material from grasses and leaves falling onto floors from thatched roofs might provide suitable sustenance for sand fleas living in the soil.

Next, elevation was shown to be positively associated with tungiasis. *Tunga* spp. tolerates high altitudes, even surviving and thriving at altitudes of over several thousand meters above sea level [50,51]. We found only one study on the relationship between elevation and tungiasis infection. This study reported no apparent spatial deviation of cases over elevation by visual comparison [12]. Taken together, there is a possibility that elevation was not a spatial driver, and other factors with a topographic distribution similar to elevation would have played a role instead in this study. Such factors would include water access, poverty, soil types, vegetation, and wildlife distributed according to elevation, some of which may be ecologically associated. As for elevation, more research is needed to account for the association of tungiasis with elevation.

Laterite, reddish-brown soil containing rich iron and aluminum oxide, can be found widely in tropical Africa [52,53], and its distribution in the African continent seems to overlap with the environmental suitability of tungiasis shown by Deka [6] (See Figure S1). A higher aluminum content in the soil was associated with a higher risk of the disease in this study. On the other hand, another characteristic of laterite, iron content in the soil, was not a significant spatial risk in model 2, which was additionally controlled for the distance to the nearest animal reserve area. This suggested that the iron content had no association with the distribution of tungiasis in the study area. One possible reason for this is the diverse composition of iron and aluminum in laterite [54]. Another possible reason is that we used the result of the model rather than actual measurements for the iron content [32].

As for aluminum, it is generally toxic to living things. Exposure to aluminum in food or medium leads to behavior abnormality, decreased fertility, growth inhibition, and a shortened lifespan in insects [55,56,57,58,59]. Considering that sand fleas’ off-host stage development occurs in soil and that larvae feed on organic matter in the environment [3,18,60], it is not impossible that a higher soil aluminum content negatively affects sand fleas’ propagation. In addition, aluminum content data might not reflect actual measurement values because they are based on a prediction by a model [32]. On the other hand, aluminum ions in the soil are strongly toxic to the plant and cause a low crop yield. A lower soil pH than 5.5, in which aluminum ions can be soluble and toxic [61,62], was found in the study area (pH 5.3–6.9), while the aluminum content in the study area (509 to 845 mg/kg) took around the median of that over the African continent (203 to 1784 mg/kg). It is thus not impossible that the environment and vegetation according to such soil may have affected sand fleas. As for the biological effect of aluminum on sand fleas, an aluminum-controlled artificial culture would be necessary for precise insight, although it is not available for sand fleas yet. Given the scarce evidence available, we suggest future studies on the relationships between soil metal content and tungiasis. Future studies might test hypotheses of the aluminum soil content and tungiasis risk on a larger scale using actual measurements of the aluminum soil content.

We found that households located closer to animal reserves had increased odds of tungiasis cases in the home. Since dogs, cats, pigs, and other livestock have been suggested to serve as animal reservoirs of sand fleas [9,11,12,13,14,17,63,64,65], wildlife in the reserve areas possibly contributed to the spreading of sand flea eggs and adults around households. Previous research in the same study area has suggested that small mammals and dogs, presumably moving in and out of the wildlife park, might bring parasites in proximity to the home, furthering the household transmission risk [17]. Wildlife themselves, or intermediary dogs and small animals that roam in and out of the bounds of the wildlife reserve, might explain why the infection risk is high in households near the park. Anecdotal reports also suggest that humans encroach on the park to forage for food and medicines and to graze animals, possibly raising the infection risk. It is unknown, however, exactly how animal habitats and human residences overlap and how likely it is that the development and infection of sand fleas occur outside living areas, where the majority of transmission is believed to occur [3,18,66]. Further studies are needed to address the dynamic ecology of sand fleas and their association with human, livestock, and wildlife interactions.

The seasonality of tungiasis [67] and the importance of moisture in the development of sand flea eggs [3] suggest the existence of an optimal range of moisture for sand fleas. However, soil moisture-related variables (NDVI and TWI) were not associated spatially with tungiasis in the study. A possible reason is that the soil moisture-related variables would have been within the tolerable ranges of sand fleas’ development, considering that the Kwale HDSS area is not so large as to have climatic differences. Another reason would be that the median value of NDVI in 2011 might have summarized the variances meaninglessly over dry and rainy seasons, although there was no choice but to use the median due to data collection over ten months in 2011. Furthermore, artificial differences in the sand flea’s habitat (i.e., ground water or drainage structure of a house) would have been more significant than natural differences.

As per our hypotheses, the combination of environmental and household factors accounted for the spatial pattern of tungiasis; however, the unexplained variance of risk remained even after adjustment. It was suggested that unmeasured spatial factors would have contributed to the spatial disparity of the disease. As noted, as a limitation, data of some known risk factors, such as socioeconomic status and animal existence at the household, were not included in the analysis. Thus, these factors might explain the residual differences in the risk of tungiasis.

There were several limitations to this study. First, as the data used in this study were collected in 2011, our results may not reflect current ecological and public health conditions. However, the results may reflect the spatial variation of the tungiasis distribution and spatial risk factors. These results can inform future data collection and research efforts to disentangle the complex ecology of tungiasis. Second, data collection on the tungiasis status took ten months across the dry and rainy seasons in 2011. Since more than half of the data were collected in rainy seasons, when transmission of sand fleas tends to be low [67], the prevalence and distribution of tungiasis would not have reflected the most infected state in the high season. Although the potentially missed cases may have affected the spatial analysis, this study identified spatial risk factors successfully. At the same time, the extended window of data collection would explain why the prevalence rate of 1.1% at the individual level in the study was remarkably lower than other studies conducted in the dry season in Kenya, which showed a 20% or more prevalence rate [7,12,16,68]. Third, the data for the socioeconomic status and livestock possession were not included in the analysis because of a five-year gap in data collection. There is a possibility that these variables, known risk factors of tungiasis, could have explained the residual geographic variation if included in the analysis. Moreover, a possibility of information bias was noticed as a potential cause of the heterogeneous distribution. In the HDSS area, of nine data collectors assigned to each area for data collection, only one covered the convex area where the center of the positive cases was located. Thus, the bias of the data collector could not be ruled out, although the distribution of tungiasis was consistent with the local awareness. A significant challenge with this study is within the method of data collection for infection status. Infection was based on self-reports or from third-party reports from other household members. In addition, the stigmatization of tungiasis in the community might have enhanced underreporting [69,70,71]. While we are unlikely to have seen false positives, there is a high possibility that some cases were missed, inflating the number of negatives. While we recognize this as a problem, we believe that the measure of household infection is sufficient and might provide a good proxy of community transmission. Despite these limitations, the present study highlights the need for further spatial studies to reveal underlying factors on tungiasis distribution.

## 5. Conclusions

In conclusion, the study showed the spatial analysis for heterogeneously distributed tungiasis using the geolocation of the household, household-based variables, and point-based ecological factors. As a result, the number of children per household, earthen floor, organic roof, elevation, aluminum content in the soil, and distance to the nearest animal reserve area were spatial risk factors at the household level in this study. Three established risk factors (children, earthen floor, and organic roof) could be promising predictors of the geographical distribution of tungiasis, whereas the roles of aluminum, elevation, and wildlife need further investigation. Considering that the variables did not fully explain the geographical disparity of tungiasis, a more complementary inclusion and consideration of variables are recommended for future work to elucidate spatial risk factors. Such spatial work will contribute to identifying high-risk populations and areas, leading to adequate control approaches.

## Figures and Tables

**Table 1:** Spatial risks used for adjustment and odds ratios in the multivariate analysis.

Household Based Variables		Tungiasis n = 7925		Mean (SD)	Model 1					Model 2				
		Negative	Positive		OR	95% CI			*p*-Value	OR	95% CI			*p*-Value
		n = 7653	n = 272											
Number of children per household				2.3 (1.9)	1.4	1.3	-	1.5	<0.01	1.4	1.3	-	1.5	<0.01
Number of elderly per household				0.28 (0.6)	1.1	0.8	-	1.4	>0.05	1.1	0.8	-	1.4	>0.05
Number of males per household				2.6 (1.8)	1.1	0.97	-	1.2	>0.05	1.1	0.97	-	1.2	>0.05
Floor	Non-earthen	1195	7		1.0					1.0				
	Earthen	6458	265		3.2	1.4	-	7.7	<0.01	3.1	1.3	-	7.6	<0.05
Wall	Non-earthen	891	6		1.0					1.0				
	Earthen	6762	266		0.5	0.2	-	1.5	>0.05	0.5	0.2	-	1.5	>0.05
Roof	Non-organic	2193	27		1.0					1.0				
	Organic	5460	245		1.7	1.1	-	2.8	<0.05	1.8	1.1	-	2.9	<0.05
Toilet	None	4235	142		1.0					1.0				
	Flush or pit latrine	3418	130		0.9	0.7	-	1.2	>0.05	0.9	0.7	-	1.2	>0.05
Normalized Difference Vegetation Index (NDVI)				157 (7.1)	1.01	0.97	-	1.04	>0.05	1.004	0.97	-	1.04	>0.05
Land cover	Grasslands	6722	234		1.0					1.0				
	Croplands	735	10		1.1	0.5	-	2.4	>0.05	1.2	0.5	-	2.8	>0.05
	Savannas	196	28		0.7	0.4	-	1.4	>0.05	0.8	0.4	-	1.5	>0.05
Topographic Wetness Index (TWI)				6.0 (1.7)	1.02	0.9	-	1.1	>0.05	1.02	0.9	-	1.1	>0.05
Elevation (10 m)				24 (8.0) *	1.2	1.1	-	1.3	<0.01	1.2	1.1	-	1.3	<0.01
Soil pH				6.0 (0.3)	0.9	0.3	-	2.2	>0.05	0.9	0.3	-	2.2	>0.05
Soil texture	Sandy clay loam	5988	267		1.0					1.0				
	Sandy loam	1665	5		0.9	0.3	-	2.5	>0.05	0.9	0.3	-	2.5	>0.05
Soil organic carbon content (g/kg)				35 (30)	0.999	0.99	-	1.003	>0.05	1.001	0.995	-	1.01	>0.05
Aluminum content of soil (10 mg/kg)				69 (7.2) *	1.1	1.03	-	1.1	<0.01	1.1	1.02	-	1.1	<0.01
Iron content of soil (10 mg/kg)				15 (1.1) *	1.2	1.01	-	1.5	<0.05	1.2	0.97	-	1.5	>0.05
Distance to the nearest animal reserve (km)				3.6 (3.4)						0.6	0.5	-	0.7	<0.01

Model 1: a generalized additive model adjusted for the number of children per household, the number of elderly per household, the number of males per household, type of floor, type of wall, type of roof, type of toilet, NDVI, land cover, TWI, elevation, soil pH, soil texture, soil organic carbon content, aluminum content in the soil, and iron content in the soil; Model 2: a generalized additive model additionally adjusted for the distance to the nearest animal reserve area; *: Note that the units of these variables are ten times larger than usual. Actual mean values are 240 m for elevation, 690 mg/kg for aluminum of soil, and 150 mg/kg for iron content of soil, as well as standard deviations; CI: confidence interval; OR: odds ratio; SD: standard deviation.

## Data Availability

The datasets generated and/or analyzed during the current study are not publicly available to secure the anonymity of the geolocation of households who participated in this study, but are available from the corresponding author on reasonable request.

## Supplementary Materials

The following are available online at https://www.mdpi.com/article/10.3390/tropicalmed7010002/s1, Table S1: Ecological data used in the analysis and its sources and details, Figure S1: Distribution of environmental suitability for tungiasis, laterite, aluminum content in soil, and iron content in soil in the African continent. R code used for analysis in this study is available online at https://github.com/hayalpaca/Spatial_risk_of_tungiasis (accessed on 10 November 2021).
