# Supplementary material for: Environmental and Household-Based Spatial Risks for Tungiasis in an Endemic Area of Coastal Kenya
Source: Trop Med Infect Dis. 2021 Dec 23;7(1):2. doi: 10.3390/tropicalmed7010002 (PMC8778305; doi:10.3390/tropicalmed7010002)
Supplement: Supplementary file 1 [file tropicalmed-07-00002-s001.zip › tropicalmed-1481851-supplementary.pdf]

Tropical Medicine and Infectious Disease

Supplementary Materials

Environmental and Household-Based Spatial Risks for Tungiasis in an Endemic Area of Coastal Kenya

Ayako Hyuga [1,2], Peter S. Larson [3,4,5], Morris Ndemwa [2,3], Sheru W. Muuo [3], Mwatasa Changoma [3], Mohamed Karama [6], Kensuke Goto [7] and Satoshi Kaneko [1,2,3,*]

1. Graduate School of Biomedical Sciences, Nagasaki University, 1-12-4 Sakamoto, Nagasaki-shi, Nagasaki 852-8523, Japan; ayako.hyuga@gmail.com (A.H.); skaneko@nagasaki-u.ac.jp (S.K.)
2. Department of Eco-Epidemiology, Institute of Tropical Medicine, Nagasaki University, 1-12-4 Sakamoto, Nagasaki-shi, Nagasaki 852-8523, Japan; morrisndmw@gmail.com
3. Nagasaki University Institute of Tropical Medicine-Kenya Medical Research Institute (NUITM-KEMRI) Project, C/O Centre for Microbiology Research, KEMRI, Nairobi P.O. Box 19993-00202, Kenya; anfangen@umich.edu (P.S.L.); sheruwanyua@yahoo.com (S.W.M.); jtasa06@gmail.com (M.C.)
4. Social Environment and Health, Survey Research Center, Institute for Social Research, University of Michigan, Ann Arbor, MI 48109, USA
5. Department of Epidemiology, School of Public Health, University of Michigan, Ann Arbor, MI 48109, USA.
6. Centre of Public Health Research, Kenya Medical Research Institute (KEMRI), Off Mbagathi Road, Nairobi P.O. Box 54840 00200, Kenya; mkarama@umma.ac.ke
7. Division of Health and Safety Sciences Education, Department of Educational Collaboration, Osaka Kyoiku University, 4-698-1 Asahigaoka, Kashiwara-shi, Osaka 582-8582, Japan; goto@cc.osaka-kyoiku.ac.jp

* Correspondence: skaneko@nagasaki-u.ac.jp

Table S1. Ecological data used in the analysis and its sources and details.

Variable	Data source	Resolution	Year/Version	Data identifier	URL
NDVI	NASA LP DAAC	250 m	2011	MOD13Q1	https://earthengine.google.com/
Land cover	NASA LP DAAC	500 m	2011	MCD12Q1	https://earthengine.google.com/
TWI	created by the authors using ASTER GDEM<br>METI/NASA (ASTER GDEM)	30 m (ASTER GDEM)	Version 3 (ASTER GDEM)	ASTGTM v003 (ASTER GDEM)	https://search.earthdata.nasa.gov/search
Elevation	JAXA	30 m	Version 3.1	AW3D30	https://www.eorc.jaxa.jp/ALOS/en/aw3d30/index.htm
Soil pH	ISRIC	250 m		SoilGrids250m 2017-03 - Soil pH in H2O 4c59ee58-a24e-4154-912e-0ff18395ac0d	https://data.isric.org/geonetwork/srv/eng/catalog.search#/metadata/4c59ee58-a24e-4154-912e-0ff18395ac0d
Soil texture	ISRIC	250 m		SoilGrids250m 2017-03 - Texture class (USDA system) f9a3a4e0-27a8-4acc-861f-26c112699c3e	https://data.isric.org/geonetwork/srv/eng/catalog.search#/metadata/f9a3a4e0-27a8-4acc-861f-26c112699c3e
Soil organic carbon content	ISRIC	250 m		SoilGrids250m 2017-03 - Soil organic carbon content (fine earth fraction) 076db4e8-11a9-4262-b6aa-cfa703a3c0af	https://data.isric.org/geonetwork/srv/eng/catalog.search#/metadata/076db4e8-11a9-4262-b6aa-cfa703a3c0af
Aluminium content in the soil	ISRIC	250m		Africa SoilGrids nutrients - Extractable Aluminium (Al) a36f7919-0d6e-4044-902c-64a74feade6b	https://data.isric.org/geonetwork/srv/eng/catalog.search#/metadata/a36f7919-0d6e-4044-902c-64a74feade6b
Iron content in the soil	ISRIC	250 m		Africa SoilGrids nutrients - Extractable Iron (Fe) 5cd5336c-2f45-4430-a9a8-312aa2095cb6	https://data.isric.org/geonetwork/srv/eng/catalog.search#/metadata/5cd5336c-2f45-4430-a9a8-312aa2095cb6
Distance to the nearest animal consevation	created by the authors				

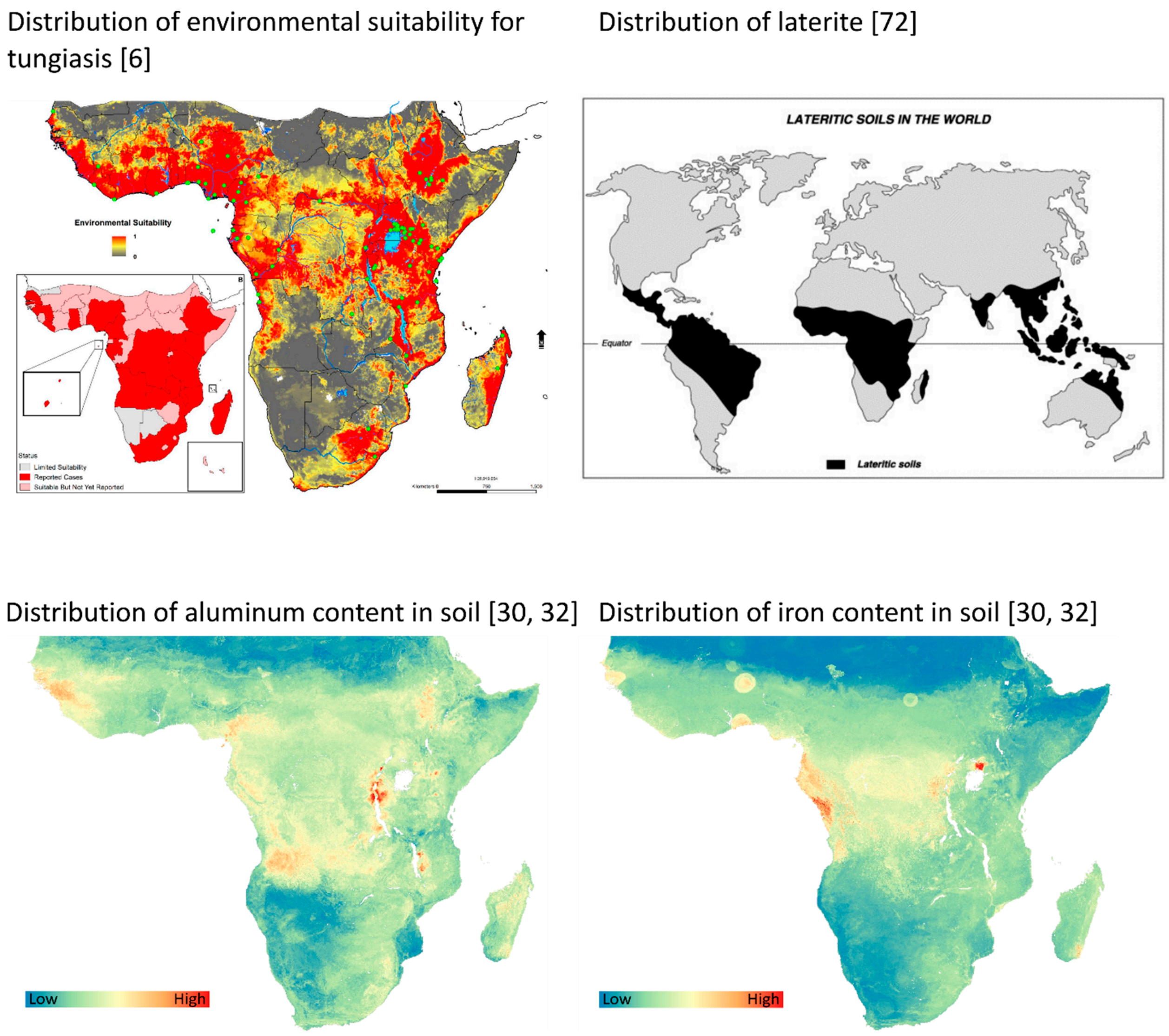

Figure S1. Distribution of environmental suitability for tungiasis, laterite, aluminum content in soil, and iron content in soil in the African continent.

References

6.Deka, M.A. Mapping the Geographic Distribution of Tungiasis in Sub-Saharan Africa. Trop. Med. Infect. Dis. 2020, 5, 122, https://doi.org/10.3390/tropicalmed5030122.
30.ISRIC Data Hub. Available online: https://data.isric.org/geonetwork/srv/eng/catalog.search#/home (accessed on 20 March 2021).
32. Hengl, T.; Leenaars, J.G.B.; Shepherd, K.D.; Walsh, M.G.; Heuvelink, G.B.M.; Mamo, T.; Tilahun, H.; Berkhout, E.; Cooper, M.; Fegraus, E.; et al. Soil nutrient maps of Sub-Saharan Africa: Assessment of soil nutrient content at 250 m spatial resolution using machine learning. Nutr. Cycl. Agroecosyst. 2017, 109, 77–102, https://doi.org/10.1007/s10705-017-9870-x.
72. Reproduced from Daniel Nahon. Altérations dans la zone tropicale. Signification à travers les mécanismes anciens et/ou encore actuels. Comptes Rendus Geoscience 2003;335(16):1109-1119. Copyright © 2003 Académie des sciences, published by Elsevier Masson SAS. All rights reserved.
